# Post-Operative Accelerated-Hypofractionated Chemoradiation With Volumetric Modulated Arc Therapy and Simultaneous Integrated Boost in Glioblastoma: A Phase I Study (ISIDE-BT-2)

**DOI:** 10.3389/fonc.2020.626400

**Published:** 2021-02-22

**Authors:** Marica Ferro, Milena Ferro, Gabriella Macchia, Savino Cilla, Milly Buwenge, Alessia Re, Carmela Romano, Mariangela Boccardi, Vincenzo Picardi, Silvia Cammelli, Eleonora Cucci, Samantha Mignogna, Liberato Di Lullo, Vincenzo Valentini, Alessio Giuseppe Morganti, Francesco Deodato

**Affiliations:** ^1^Radiation Oncology Unit, Gemelli Molise Hospital – Università Cattolica del Sacro Cuore, Campobasso, Italy; ^2^Medical Physics Unit, Gemelli Molise Hospital – Università Cattolica del Sacro Cuore, Campobasso, Italy; ^3^Radiation Oncology, IRCCS Azienda Ospedaliero-Universitaria di Bologna, Bologna, Italy; ^4^DIMES, Alma Mater Studiorum Bologna University, Bologna, Italy; ^5^Radiology Unit, Gemelli Molise Hospital – Università Cattolica del Sacro Cuore, Campobasso, Italy; ^6^Medical Oncology Unit, Gemelli Molise Hospital – Università Cattolica del Sacro Cuore, Campobasso, Italy; ^7^Fondazione Policlinico Universitario A. Gemelli, IRCCS, UOC di Radioterapia, Dipartimento di Scienze Radiologiche, Radioterapiche ed Ematologiche, Roma, Italy; ^8^Istituto di Radiologia, Università Cattolica del Sacro Cuore, Roma, Italy

**Keywords:** glioblastoma multiforme, volumetric modulated arc therapy, simultaneous integrated boost, adjuvant treatment, temozolomide

## Abstract

**Background:**

Glioblastoma Multiforme (GBM) is the most common primary brain cancer and one of the most lethal tumors. Theoretically, modern radiotherapy (RT) techniques allow dose-escalation due to the reduced irradiation of healthy tissues. This study aimed to define the adjuvant maximum tolerated dose (MTD) using volumetric modulated arc RT with simultaneous integrated boost (VMAT-SIB) plus standard dose temozolomide (TMZ) in GBM.

**Methods:**

A Phase I clinical trial was performed in operated GBM patients using VMAT-SIB technique with progressively increased total dose. RT was delivered in 25 fractions (5 weeks) to two planning target volumes (PTVs) defined by adding a 5-mm margin to the clinical target volumes (CTVs). The CTV_1_ was the tumor bed plus the MRI enhancing residual lesion with 10-mm margin. The CTV_2_ was the CTV_1_ plus 20-mm margin. Only PTV_1_ dose was escalated (planned dose levels: 72.5, 75, 77.5, 80, 82.5, 85 Gy), while PTV_2_ dose remained unchanged (45 Gy/1.8 Gy). Concurrent and sequential TMZ was prescribed according to the EORTC/NCIC protocol. Dose-limiting toxicities (DLTs) were defined as any G ≥ 3 non-hematological acute toxicity or any G ≥ 4 acute hematological toxicities (RTOG scale) or any G ≥ 2 late toxicities (RTOG-EORTC scale).

**Results:**

Thirty-seven patients (M/F: 21/16; median age: 59 years; median follow-up: 12 months) were enrolled and treated as follows: 6 patients (72.5 Gy), 10 patients (75 Gy), 10 patients (77.5 Gy), 9 patients (80 Gy), 2 patients (82.5 Gy), and 0 patients (85 Gy). Eleven patients (29.7%) had G1-2 acute neurological toxicity, while 3 patients (8.1%) showed G ≥ 3 acute neurological toxicities at 77.5 Gy, 80 Gy, and 82.5 Gy levels, respectively. Since two DLTs (G3 neurological: 1 patient and G5 hematological toxicity: 1 patient) were observed at 82.5 Gy level, the trial was closed and the 80 Gy dose-level was defined as the MTD. Two asymptomatic histologically proven radionecrosis were recorded.

**Conclusions:**

According to the results of this Phase I trial, 80 Gy in 25 fractions accelerated hypofractionated RT is the MTD using VMAT-SIB plus standard dose TMZ in resected GBM.

## Introduction

Glioblastoma Multiforme (GBM) is the most common primary brain tumor in adults (1). The standard of care is surgical resection followed by radiation therapy (RT) plus concurrent and adjuvant Temozolomide (TMZ) (2). However, the GBM prognosis remains poor being 5.6% the 5-year overall survival (OS) rate and 10–15 months the median survival (3, 4).

Since the ‘70s, 60 Gy in 2 Gy per fraction has been the standard postoperative RT dose, outside clinical trials (5, 6). Higher doses could be more effective but also associated with an increased risk of healthy tissues damage. However, significant technological advances have been achieved in the past decades in brain tumors RT planning and delivery. In fact, intensity modulated RT (IMRT) and volumetric modulated arc therapy (VMAT) lead to improved dose conformality to the target. Moreover, sparing of the surrounding organs at risk (OARs) promoted the delivery of an accelerated-hypofractionated simultaneous integrated boost (SIB) (7, 8).

More generally, hypofractionated RT resulted feasible in GBM patients with reduced overall treatment time and higher biologically equivalent dose (9–12). Indeed, both decreased tumor repopulation and increased cells death are radiobiological advantages of accelerated-hypofractionated regimens (13). Moreover, a shorter treatment duration may improve patients’ comfort and reduce treatment-related costs. Therefore, hypofractionated RT schedules were increasingly used in dose-escalation studies to test the possibility of overcoming the intrinsic GBM radiation-resistance (14–18).

Our group reported the feasibility of postoperative IMRT-SIB up to 70 Gy in 25 fractions in GBM (15, 17). Based on this result and on the growing experience in VMAT-SIB in other settings (19, 20), we designed a phase I trial to define the maximum tolerated dose (MTD) of adjuvant VMAT-SIB plus TMZ. Here we report the results of this trial.

## Materials and Methods

### Inclusion Criteria

The inclusion criteria were as follows: 1) histologically-proven GBM (World Health Organization 2007); 2) age ≥ 18 and ≤ 85 years; 3) Eastern Cooperative Oncology Group (ECOG) performance status ≤ 3; 4) estimated survival ≥ 3 months; 5) normal organ and bone marrow function (white blood cell count > 3,000/mm^3^; hemoglobin > 9 g/dl; platelets > 100,000/mm^3^). All patients underwent a first evaluation with clinical history and physical examination. Patients with previous brain irradiation, multifocal GBM, other malignancy (except cervical carcinoma *in situ* and non-melanoma skin cancer), and pregnant or breast-feeding were excluded.

### Study Design and End Point

This prospective phase-I trial (ISIDE BT-2) was approved by the Catholic University Institutional Review Board (#42/07-29-2015) and patients signed a written informed consent. Patients were enrolled in subsequent cohorts of three subjects with progressively higher boost dose as reported in **Table 1**. The primary end point was to define the MTD considered as the dose-level below the one with dose limiting toxicity (DLT) recorded in at least one third of patients. Any acute G ≥ 3 non-hematological adverse event or any acute G ≥ 4 hematological toxicity or any late G ≥ 2 toxicity was defined as DLT (21). If no DLTs were recorded, patients were enrolled at the next dose level provided that all patients in the cohort had been followed for at least six months. If a DLT occurred in ≥ two patients, the study was closed and the previous dose level was considered as the MTD. If a DLT was recorded in one patient, further enrollment up to a minimum of six patients (with ≥ 6 months follow-up) was required at the same dose-level. In this case, the study continued as follows: a) if DLT occurred in one patient, the subsequent patients were enrolled in the next cohort; b) if DLT occurred in more than two patients, the study was closed and the MTD was defined as the previous dose level; c) if DLT occurred in two patients, the study was closed with the MTD defined as the same dose level. A total SIB-boost dose of 85 Gy in 25 fractions was considered as the highest dose level in the study design.

**Table 1:** Dose cohorts and dose escalation levels.

Radiation total dose/fraction size					
Planned patients	Treated patients	Dose level	PTV2 dose/fractionation	PTV1 dose/fractionation (BED_α/β=5.6 Gy_)	Concurrent temozolomide
3	6	I	45 Gy/1.8 Gy	72.5 Gy/2.9 Gy (98.6 Gy)	75 mg/m^2^ daily
3	10	II	45 Gy/1.8 Gy	75.0 Gy/3.0 Gy (103.7 Gy)	75 mg/m^2^ daily
3	10	III	45 Gy/1.8 Gy	77.5 Gy/3.1 Gy (109.0 Gy)	75 mg/m^2^ daily
3	9	IV	45 Gy/1.8 Gy	80.0 Gy/3.2 Gy (114.3 Gy)	75 mg/m^2^ daily
3	2	V	45 Gy/1.8 Gy	82.5 Gy/3.3 Gy (119.7 Gy)	75 mg/m^2^ daily
3	0	VI	45 Gy/1.8 Gy	85.0 Gy/3.4 Gy (125.2 Gy)	75 mg/m^2^ daily

BED, Biologically Equivalent Dose; PTV, planning target volume.

### Radiotherapy

#### Treatment Planning

Treatment simulation and OARs contouring were previously described (15). An IMRT Reinforced Thermoplastics™ mask was used for patient immobilization. The head was held using a support (Uni-frame^®^ Tilting Baseplate, CIVCO Medical Solutions, IA, US) providing a tilt movement able to misalign the brain from the eyes. CT-simulation scans (3 mm thickness at 3 mm interval) were acquired from the vertex up to the lower margin of the second cervical vertebra. Patients underwent multiparametric (spectroscopy, diffusion, and perfusion) gadolinium enhanced MRI four weeks after surgery. MRI scans were co-registered with the planning CT-simulation scans to optimize the delineation of target volumes and OARs. The gross tumor volume (GTV) was defined as the resection cavity, any residual disease, and contrast-enhanced areas in T1-weighted MRI. The clinical target volume 1 (CTV_1_) was defined as the GTV plus 10-mm margin (only in the brain), including any microscopic tumor spread. The CTV_2_ was defined by adding a 20-mm isotropic margin to the CTV_1_. Subsequently, the CTV_2_ was manually edited to exclude the extracerebral tissues and in particular the OARs. For set-up uncertainties, an isotropic 5-mm margin was added to CTV_1_ and CTV_2_ to define the planning target volumes (PTV_1_ and PTV_2_, respectively). VMAT plans were calculated using the “dual arc” feature, based on two partial coplanar arcs (6-MV nominal photon energy). Treatment plans were calculated with the OncentraMasterPlan^®^ Treatment Planning System v. 4.1 (Nucletron BV, Veenendaal, The Netherlands) based on ICRU 83 recommendations. Dose/volume constraints and quality assurance procedures have been previously detailed (15, 17). All treatment plans were calculated by a senior physicist (SaC) and reviewed for target coverage and dose/volume constraints by a radiation oncologist expert in brain tumors RT (MaF).

#### Treatment delivery

VMAT-SIB was delivered in 25 fractions using an Elekta Precise linear accelerator (Elekta Ltd., Crawley, UK). Only PTV_1_ dose was escalated (planned dose escalation: 72.5 Gy, 75.0 Gy, 77.5 Gy, 80.0 Gy, 82.5 Gy, and 85.0 Gy) while maintaining the same dose to PTV_2_ (45.0 Gy in 1.8 Gy/fraction). The biologically effective dose (BED) corresponding to the different dose levels is shown in **Table 1**. The BED was calculated according to the formula:

BED=nd[1+d/(α/β)]−γ(T−Tk)/α

where n = number of fraction, d = fractionation dose, T = overall treatment time, Tk = time at which repopulation begins after treatment, γ = effective tumor-cell repopulation rate: γ = ln 2/Td, where Td = potential doubling time (22). Based on Qi et al. estimation of radiobiological parameters of brain tumor (23), we used the following values for BED calculation: α = 0.04, α/β ratio = 5.6 Gy, potential doubling time = 50 days, and kickoff time for accelerated repopulation = 0 days.

### Chemotherapy

Concurrent TMZ protocol was 75 mg/m²/day, 7 days per week, for the entire RT duration (2). Four weeks after chemoradiation, patients received up to 12 cycles of adjuvant TMZ (150-200 mg/m²/day, 5 days every 28 days). TMZ was discontinued in case of progressive disease or G ≥ 3 toxicity. Dexamethasone (2.25 mg/day) was prescribed to all patients during RT. This dosage was not reduced in patients taking higher doses before treatment and it was increased in case of neurotoxicity.

### Toxicity Assessment

Toxicity was classified in terms of grade, type, and possible relationship to the treatment. Acute toxicity was scored using the Radiation Therapy Oncology Group (RTOG) criteria and late toxicity was assessed based on the RTOG/European Organization for Research and Treatment of Cancer (RTOG/EORTC) scale (21). Acute toxicities were defined as those occurring within three months from RT. Adverse events recorded at least three months after the start of radiation therapy were defined as late toxicities.

### Patients Follow-Up and Response Criteria

Patients were evaluated three weeks after treatment completion and then every two months with clinical examination and blood tests. A contrast-enhanced multiparametric brain MRI was performed 45 days after RT completion and then every two months. Clinical response was evaluated based on the RECIST criteria in patients with macroscopic residual disease after surgery (24). In case of suspected pseudoprogression, a 6-[18F]-L-fluoro-L-3,4-dihydroxyphenylalanine-PET/CT was performed. In case of progressive disease in the brain, patients were considered for salvage treatment on a case-by-case basis (re-operation, second-line chemotherapy or re-irradiation).

### Statistical Analysis

The Kaplan-Meier method (25) was used to calculate progression-free survival (PFS) and OS curves. PFS was defined as the time between surgical resection and disease progression while OS as the time between surgery and death from any cause. Statistical analysis was performed using SYSTAT, version 11.0 (SPSS, Chicago, IL).

## Results

Between January 2012 and November 2018, 37 patients were enrolled in the trial. Molecular data were available only for a minority of patients: 11 of 37 patiens had isocitrate dehydrogenase 1 (IDH) wild-type while p53 was expressed in 9 of 11 patients.

Dose cohorts and patient characteristics are detailed in **Tables 1** and **2**, respectively. Median follow-up was 12 months (range: 2-67 months).

**Table 2:** Patient and tumor characteristics.

Patients	N (%)
Total	37
Gender	
Male	21 (56.8%)
Female	16 (43.2%)
Age (years)	
Median	59
Range	36–82
ECOG performance status	
0	15 (40.5%)
1	14 (37.9%)
2	4 (10.8%)
3	4 (10.8%)
Surgery	
Gross total resection	10 (27.0%)
Partial resection	27 (73.0%)
RTOG-RPA class*	
III	7 (18.9%)
IV	26 (70.3%)
V	4 (10.8%)
Planning target volume 1 (cc)	
Median	175.8
Range	53.9–292.9
Planning target volume 2 (cc)	
Median	451.9
Range	194.0–620.5
Tumor site	
Frontal	11 (29.7%)
Parietal	11(29.7%)
Temporal	4(10.8%)
Frontoparietal	2 (5.5%)
Parietotemporal	9 (24.3%)
Side	
Right	16 (43.2%)
Left	21 (56.8%)
MGMT promoter status	
Not available	21 (56.7%)
Methylated	9 (24.3%)
Not methylated	7 (19.0%)

ECOG, Eastern Cooperative Oncology Group; RPA, recursive partitioning analysis (Curran 1993): RTOG, Radiation Therapy Oncology Group; MGMT, O6-methylguanin-

DNA-methyltransferase.

### Maximum Tolerated Dose

One patient treated at level I (72.5 Gy, 2.9 Gy/fraction) developed G4 hematologic toxicity at the end of chemoradiation. Therefore, three more patients were enrolled in the same cohort and no other DLTs were recorded. At dose level II (75.0 Gy, 3 Gy/fraction), one out of three patients showed G4 hematologic toxicity resulting in permanent discontinuation of TMZ. Therefore, also this cohort was expanded to six patients. Before reaching six months of observation of the planned patients, four more subjects were treated at dose level II (total: 10 patients). Since no other DLT were recorded, the dose was escalated to level III (77.5 Gy, 3.1 Gy/fraction). One out of the three patients in this cohort presented DLT (severe neurological toxicity) and died due to toxicity worsening before starting of adjuvant TMZ. This G5 adverse event required the enrollment of three more subjects. For the same reason as in the second cohort (the need for adequate follow-up), a total of 10 patients were enrolled at this dose level without other recorded DLT. Nine patients were enrolled at level IV (80 Gy, 3.2 Gy/fraction) due to one case of G3 seizures and need of adequate follow-up. Two patients were enrolled in the subsequent cohort at level V (82.5 Gy, 3.3 Gy/fraction) and both showed severe toxicity. The first patient developed severe seizures requiring hospitalization one month after RT while the second one discontinued chemoradiation due to severe hematological toxicity. Before starting of adjuvant TMZ, the latter patient died of myelosuppression worsening. Since two DLTs were observed in two patients at level V the trial was closed and level IV (80 Gy, 3.2 Gy/fraction) was considered as the MTD (**Table 3**).

**Table 3:** Acute toxicity (RTOG scale).

Dose Levels*						
Toxicity	Grade	I: 72.5 Gy	II: 75.0 Gy	III: 77.5 Gy	IV: 80.0 Gy	V: 82.5 Gy
Neurological	0 1-2 3-5	4 2 0	7 3 0	6 3 **1**	5 3 1	1 0 1
Eye	0 1-2 3-5	6 0 0	10 0 0	10 0 0	9 0 0	2 0 0
Skin	0 1-2 3-5	0 6 0	0 10 0	0 10 0	0 9 0	0 2 0
Hemoglobin	0 1-2 3-5	5 0 1	10 0 1	10 0 0	9 0 0	1 0 **1^#^**
WBC	0 1-2 3-5	5 0 1	10 0 1	10 0 0	9 0 0	1 0 **1^#^**
Neutrophils	0 1-2 3-5	5 0 1	9 0 1	10 0 0	9 0 0	1 0 **1^#^**
Platelets	0 1-2 3-5	5 0 1	9 0 1	10 0 0	9 0 0	1 0 **1^#^**

*Data relative to dose level of 85 Gy are not shown, due to lack of accrual. Numbers in bold represent the G5 toxicities; **^#^**same patient.

### Treatment Compliance

**Figure 1:**
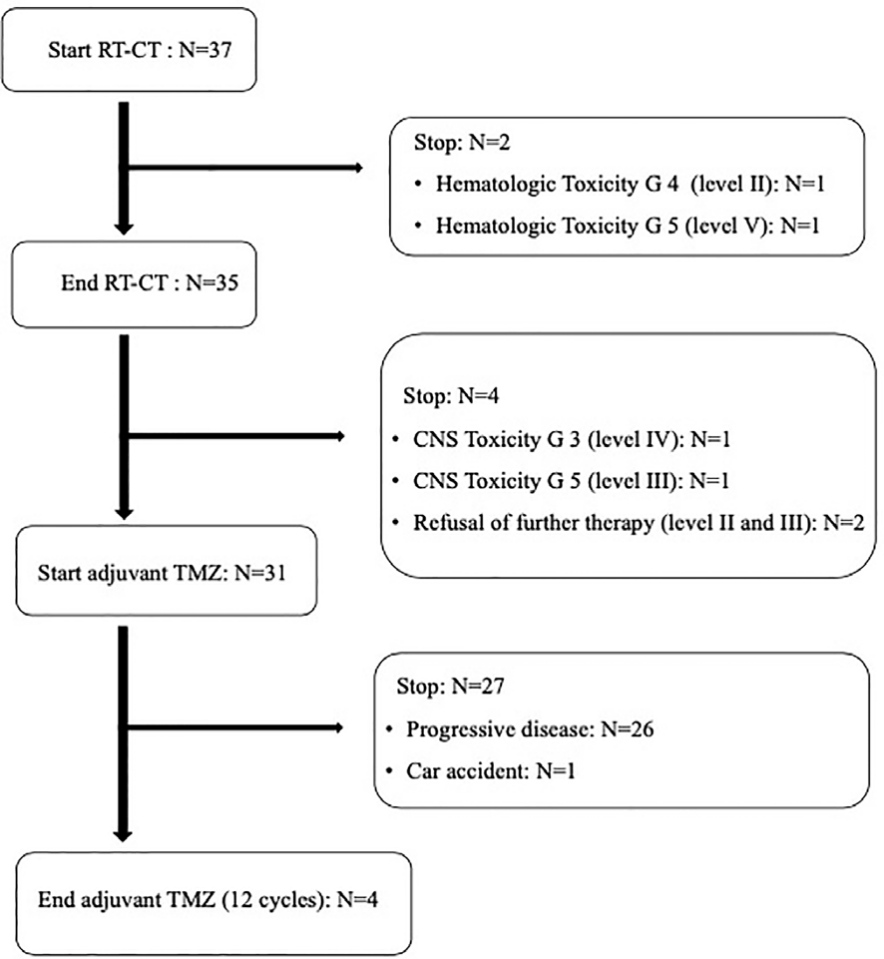
Overall treatment compliance.

All patients received concurrent TMZ, but in two patients (at dose level II and V, respectively) chemoradiation was permanently stopped (after 20 and 22 fractions, respectively) due to hematological toxicity. For the same reason, these patients did not receive adjuvant TMZ (**Figure 1**). In addition, another patient interrupted chemoradiation for three days due to hematological toxicity. Only 31 out of 35 patients potentially amenable to adjuvant TMZ started chemotherapy. In fact, two patients refused chemotherapy and two patients were unable to start TMZ due to severe neurological toxicity (G ≥ 3) (**Figure 1**). Moreover, only four patients completed the prescribed 12 cycles of adjuvant TMZ. In fact, 27 patients discontinued adjuvant chemotherapy after 2-11 TMZ cycles due to disease progression in 26 patients and early death not related to treatment and disease in one patient (**Figure 1**).

### Toxicity

DLTs were only represented by neurological and hematological toxicities (**Table 3**). Grade ≥ 3 neurological toxicity occurred in three patients (8.1%). One patient died after worsening of neurological symptoms and two patients had multiple seizures despite medical intervention. Grade ≥ 3 hematological toxicity occurred in three patients (8.1%, two females and one male). One patient died two months after chemoradiation due to prolonged myelosuppression and worsened general conditions. Two patients had G3-4 anemia, thrombocytopenia, and leucopenia which prevented the start or caused the interruption of adjuvant chemotherapy. Grade 1 or 2 neurological findings, mainly nausea and headache, were recorded in seven (18.9%) and four (10.8%) patients, respectively. No patient had G1-2 hematological toxicity while all patients had G1-2 skin toxicity, mainly epilation or mild erythema in the irradiated site. No patient showed ocular toxicity despite the frontal or frontoparietal site of the irradiated lesion in 13 subjects.

No patient reported or showed symptoms related to severe late toxicity. Three patients (dose level II, III, and IV) reported mild (G1) headache during the follow-up. However, two cases of radionecrosis (5.4%) were histologically proven at 10 and 52 months after chemoradiation (dose level I and IV).

### Outcomes

Thirty-three out of 37 patients (89.2%) underwent MRI six-seven weeks after chemoradiation and all of them showed stable disease compared to pre-RT evaluation. Four patients were not evaluable for clinical response due to patient’s refusal (two) or poor general conditions due to unresolved toxicity (two). Thirty-two patients had local progressive disease in the high dose region (central recurrence) while no out-of-field relapse was recorded. Twenty patients were amenable for salvage therapy: four and 10 patients underwent stereotactic RT or salvage chemotherapy, respectively, while six patients were re-operated. In the latter, two radio-necrosis and four local recurrences were histologically proven. Median PFS and OS were 10 and 17 months, respectively. Actuarial 1- and 2-year PFS was 27% and 8%, respectively, while 1- and 2-year OS was 67 and 22%, respectively.

## Discussion

To the best of our knowledge, this study represents the first phase I dose-escalation trial on postoperative VMAT-SIB combined with TMZ in GMB patients. In our previous studies, based on the IMRT-SIB technique (15, 17), we did not reach the MTD up to the dose of 70 Gy in 25 fractions (2.8 Gy/fraction). In the present study, the MTD of postoperative VMAT-SIB plus standard TMZ in operated GMB resulted 80 Gy in 25 fractions (3.2 Gy/fraction). Unfortunately, it is a study reporting no improved outcome and a trend toward more hematologic and neurologic toxicity.

Some limitations may be ascribed to the study. First of all, the use of a classic dose-escalation design (3 + 3) can correctly assess short-term but not long-term tolerability. For example, Tsien’s et al., in their dose-escalation study, used the time-to-event continual reassessment method, a Bayesian dose-finding design to address the issue of long observation time and early patient drop-out (26). We partially mitigated this limitation in our trial by requiring the observation of the three patients included in a cohort for at least six months. Also the small sample size of our study does not provide adequate information on the risk of late toxicity. For this reason, a phase I-II trial on a larger patient population treated at the MTD defined in this study is ongoing. Furthermore, the definition of DLT was based on rather obsolete toxicity scales (RTOG and EORTC-RTOG). These choices resulted from the intentional continuity of this study with our previous trials (15, 17) which began in 2005. Moreover, the study’s inclusion period was relatively long (6 years) due to the small Italian region where we work that did not allow us a faster accrual of GBM patients. Last, in the classification of tumor relapses, we used only the in-field and out-of-field categories, unlike other authors who also considered the “central” and “marginal” categories.

Beyond these limitations, our study was able to define the MTD of adjuvant RT in GBM, unlike other studies. In fact, in several phase I trials, no DLT was registered and therefore the MTD was not reached (11, 12, 16, 18, 27). Only the study of Tsien et al. (26) defined 75 Gy (2.5 Gy/fraction) as the MTD, a value lower compared to our trial (80 Gy, 3.2 Gy/fraction). This discrepancy could result from the different design of the two studies, as described above.

In our trial, G ≥ 3 neurological toxicity occurred in 8.1% of patients. As expected, these figures are higher than those (0.7%) reported by Stupp et al. in the EORTC/NCIC trial based on the delivery of 60 Gy in 2 Gy/fraction (2). Moreover, our severe neurological toxicity rate is similar to those reported in other dose escalation trials (10.5% -19.0%) (16, 26, 27), despite the use of higher dose/fractionation. The use of the VMAT technique could be an explanation of this effect. However, it should be noted that no cases of severe neurological toxicity were recorded in some of the other dose-escalation studies (11, 12, 18). The explanations may be different, such as the small GTV to PTV margins (0.5 cm) in the study by Chen et al. (11), the use of a standard dose (60 Gy) even if slightly hypofractionated (3 Gy/fraction) in the study by Jastaniyah et al. (12), and finally the small sample size (only 9 patients enrolled) in the study by Truc et al. (18).

Someone could argue that the doses and the volumes (margins) used in the study may be not optimal. It is complex to be able to make comparisons with other studies like Stupp’s one and extrapolate conclusions. The choice we pursued was to reduce the prophylactic dose and greatly increase the CTV1 dose. We started from the assumption that patterns of failure studies have shown that 80–90% of recurrences occur within 2–3 cm of the surgical cavity. Furthermore, multiple series showed that patients who received a total brain dose of 60 Gy still failed within the highest dose region. Moreover, we prudentially set the margins trying to encompass any microscopic tumor spread. One centimeter around the GTV in dose escalation volume may have contributed to the increase in especially neurological toxicity. However, a clear direction on this issue is still lacking and the standard margins for patients with GBM are likely to continue to evolve over time. The ability to utilize MRI (perfusion) and PET data in target delineation, i.e. the next generation imaging would probably have allowed us to define narrower margins around CTV1, however, the present dose escalation trial was conceived in 2005 when the novel imaging modalities were not widely available. For the sake of continuity, we followed the same modality of target delineation adopted in our previous trials (15, 17).

Our results and those from other studies confirm the impact of use and type of chemotherapy concurrent to dose-escalated RT on hematological toxicity. Indeed, the rate of G ≥ 3 blood/bone marrow complications recorded in our study (8.1%) was similar to that observed by Jastaniyah et al. (12) (8.0%) who used concurrent TMZ as in our trial. Instead, Tsien et al. (27), who combined RT with carmustine, reported a 44.5% rate of severe hematological complications. On the contrary, Monjazeb et al. (16) treated their patients with RT alone without recording any case of G ≥ 3 hematological toxicities.

The hematological toxicity recorded in our study was only severe (G1-2: 0%; G3-5: 8.1%). This data would confirm the hypothesis that this type of complication is not due to a simple toxic effect on hematopoietic cells but is based on an idiosyncratic mechanism linked to genetic factors. In any case, this high risk of severe and even fatal complications, as reported in our and Tsien’s et al. (26) experiences, suggests the need for close monitoring of bone marrow function in order to promptly prevent possible complications.

In terms of disease control, the results of our trial are rather discouraging. Although the use of the VMAT-SIB technique allowed the delivery of BED values higher compared to the previously published studies, we recorded an in-field relapse rate of 100% in evaluable patients.

This result confirms the widespread skepticism about the potential role of dose escalation in GBM. Only a few studies suggested an improvement in the outcome with higher than standard doses (28, 29), while most evidence showed lack of improved outcomes (30–33). This would explain the trend toward a progressive reduction of higher than the standard dose RT recorded in the USA (31).

However, considering our and the other phase I studies on high dose RT combined with concurrent TMZ (11, 12, 18, 26), it should be noted that they consistently reported a higher median survival (15.7–22.4 months) compared to RT plus TMZ arm of the EORTC/NCIC trial (14.6 months).

New treatment options for GBM have become available in recent years including immunotherapy, targeted therapies, radiosensitizers, novel irradiation modalities, and tumor-treating fields (34). It can be hypothesized that the combination with some of these innovative therapies may improve the results of standard chemoradiation. For example, the study by Stupp et al. recorded an improved survival combining tumor-treating fields to maintenance TMZ compared to the standard protocol (35).

Furthermore, it is possible that new combined modality treatments can exploit the effect of higher than standard doses. Studies to test this hypothesis could employ the recommended doses defined in our and in the Tsien’s et al. trials (26).

In addition to studies on new treatments combinations, further analyzes would be warranted to improve the dismal results of GBM treatment. Concerning the new irradiation modalities, Matsuda et al. recently reported overall survival improvement in using proton beams with standard fractionation or hypofractionation with concomitant boost technique (36). Moreover, the use of RT dose escalation could be of benefit in specific subgroups of patients, while, conversely, other groups of patients may be more prone to treatment-induced toxic effects. Therefore, the development of predictive models could allow to identify patients in whom the delivery of high doses is justified and of patients at high risk of toxicity where treatment de-escalation could be preferable. Finally, future studies on high dose RT should include the assessment of the impact on quality of life. This topic deserves to be carefully considered given the poor prognosis of these patients and therefore the substantially palliative meaning of RT in this setting.

## Data Availability Statement

The raw data supporting the conclusions of this article will be made available by the authors, without undue reservation.

## Ethics Statement

The studies involving human participants were reviewed and approved by Catholic University Institutional Review Board. The patients/participants provided their written informed consent to participate in this study.

## Author Contributions

VV, AM, and FD were the guarantors of integrity of the entire study and contributed to study concepts and design. The literature research as well as the manuscript writing were carried on by MaF, MiF and GM. Data and statistical analysis were conducted by GM, SaC, and CR. MaF, AR, MaB, VP, EC, SM, LL, and SiC contributed to clinical studies whereas the manuscript was edited by MiB, AM, FD and VV. All authors contributed to the article and approved the submitted version.

## Conflict of Interest

The authors declare that the research was conducted in the absence of any commercial or financial relationships that could be construed as a potential conflict of interest.
